# Decreased expression of GRAF1/OPHN-1-L in the X-linked alpha thalassemia mental retardation syndrome

**DOI:** 10.1186/1755-8794-3-28

**Published:** 2010-07-06

**Authors:** Vincenza Barresi, Angela Ragusa, Marco Fichera, Nicolò Musso, Lucia Castiglia, Giancarlo Rappazzo, Salvatore Travali, Teresa Mattina, Corrado Romano, Guido Cocchi, Daniele F Condorelli

**Affiliations:** 1 Laboratorio sui Sistemi Complessi, Scuola Superiore di Catania, University of Catania, Catania, Italy; 2 Section of Biochemistry and Molecular Biology, Department of Chemical Sciences, University of Catania, Catania, Italy; 3 Department of Biomedical Sciences, University of Catania, Catania, Italy; 4 Department of Animal Biology, University of Catania, Catania, Italy; 5 Department of Pediatrics, University of Catania, Catania, Italy; 6 ICPPN, Bologna, Italy; 7 IRCCS OASI Maria SS Troina, Catania, Italy; 8 UOC Diagnosi Prenatale Laboratorio di Genetica Umana AOU Policlinico-Vittorio Emanuele, Catania, Italy

## Abstract

**Background:**

ATRX is a severe X-linked disorder characterized by mental retardation, facial dysmorphism, urogenital abnormalities and alpha-thalassemia. The disease is caused by mutations in ATRX gene, which encodes a protein belonging to the SWI/SNF DNA helicase family, a group of proteins involved in the regulation of gene transcription at the chromatin level. In order to identify specific genes involved in the pathogenesis of the disease, we compared, by cDNA microarray, the expression levels of approximately 8500 transcripts between ATRX and normal males of comparable age.

**Methods:**

cDNA microarray was performed using total RNA from peripheral blood mononuclear cells of ATRX and normal males. Microarray results were validated by quantitative real-time polymerase chain reaction.

**Results:**

cDNA microarray analysis showed that 35 genes had a lower expression (30-35% of controls) while 25 transcripts had a two-fold higher expression in comparison to controls. In the microarray results the probe for oligophrenin-1, a gene known for its involvement in mental retardation, showed a decreased hybridization signal. However, such gene was poorly expressed in blood mononuclear cells and its decrease was not confirmed in the quantitative real-time RT-PCR assay. On the other hand, the expression of an homologous gene, the GTPase regulator associated with the focal adhesion kinase 1/Oligophrenin-1-like (GRAF1/OPHN-1-L), was relatively high in blood mononuclear cells and significantly decreased in ATRX patients. The analysis of the expression pattern of the GRAF1/OPHN-1-L gene in human tissues and organs revealed the predominant brain expression of a novel splicing isoform, called variant-3.

**Conclusions:**

Our data support the hypothesis of a primary role for altered gene expression in ATRX syndrome and suggest that the GRAF1/OPHN-1-L gene might be involved in the pathogenesis of the mental retardation. Moreover a novel alternative splicing transcript of such gene, predominantly expressed in brain tissues, was identified.

## Background

The XNP/ATRX gene encodes a 2492 amino acid chromatin-associated protein which bears, at the N-terminus, a region (encoded by exons 8-10) named ATRX-DNMT3-DNMT3L (ADD) domain and containing a N-terminal GATA-like zinc finger, a plant homeodomain finger and a long C-terminal that pack together to form a single globular domain [1,2]. At the C-terminus is present a helicase/ATP domain (encoded by exons 18-31) formed by seven conserved "helicase" motifs found in DNA-stimulated ATPases and DNA helicases of the SNF2/SWI2 protein family [3-6]. SWI/SNF [Switching defective (SWI) and Sucrose nonfermenting) (SNF)] complexes function as global gene regulators, altering the chromatin structure and changing the accessibility of transcription factor to DNA in a subset of specific genes [7].

Mutations in the XNP/ATRX gene, located in Xq13.3, are associated with × linked mental retardation syndromes, the best known being alpha thalassaemia with mental retardation (ATRX, MIM 301040) [3,4,8-12]. Previous studies have shown that ATRX mutations are predominantly found within the zinc-finger or the helicase domain and result in comparable clinical manifestations[11,12].

Several lines of evidence suggest that XNP/ATRX is involved in gene expression regulation via chromatin remodelling through the following mechanisms: 1) its association with the human EZH2 protein [5,13], a human homologue of the Enhancer of Zeste *Drosophila *gene involved in the regulation of homeotic gene expression through chromatin remodelling; 2) its interaction with the heterochromatin protein HP1 [14]; 3) the abnormalities of DNA methylation profiles of repetitive elements induced by ATRX mutations [12]; 4) the nuclear localization and the close association with pericentromeric heterochromatin during mitosis [13,15]; 5) the documented DNA-binding activity of the zinc finger domain [6]; 6) the co-localization with the transcription co-activator Daxx in promyelocytic leukaemia nuclear bodies [16].

To date the cellular mechanism(s) underlying the ATRX syndrome remain unknown. Despite the rarity of this syndrome the identification of involved genes can supply useful data for the general understanding of the molecular mechanisms responsible for mental retardation.

In the present work we have applied the cDNA microrray technique as an exploratory preliminary tool with the intent to identify alterations of gene expression that might provide useful hints to the pathogenetic mechanism of the ATRX syndrome. Validation of microarray results was performed by quantitative real-time polymerase chain reaction (qRT-PCR analysis). Although the analysis was performed on peripheral blood cells, significant changes in the expression profile were revealed. Moreover, GTPase regulator associated with the focal adhesion kinase 1/Oligophrenin-1-like (GRAF1/OPHN-1-L), one of the gene showing altered expression level in ATRX, is potentially involved in Central Nervous System (CNS) function and in mental retardation and we report the identification of a novel alternative transcript and its tissue and brain distribution.

## Methods

### Clinical findings and ATRX gene mutations

Three male patients, ATRX-1 (age 12 years), ATRX-2 (age 12 years) and ATRX-3 (age 14 yrs), presenting peculiar features of the ATRX syndrome, were analysed in the present study after informed consent by parents or ligal guardians. All patients showed a profound mental retardation, microcephaly, facial dysmorphisms, and short stature (all below the 3^rd ^centile). Clinical findings and ATRX gene mutations of each patient were previously described [9,17].

### Peripheral blood mononuclear cell isolation

Peripheral blood mononuclear (PBM) cells were isolated from 5 ml of EDTA (K_3_) [K_3_E] blood by Ficoll gradient procedure. The whole blood was diluted with Hanks's solution (volume ratio 1:1) (Life Technologies, Paisley, Scotland) and was added to tube with Ficoll Separating Solution (volume ratio 1:1) (BIOCHROM KG, Berlin). After centrifugation for 35 min a 400 *g*, the mononuclear cells containing layer was carefully pipetted away and resuspended in Phosphate Buffer Saline (PBS, pH 7.4). The cellular pellet was washed in PBS solution two times.

### RNA extraction and cDNA synthesis

Total RNA was extracted as described by Chomczynski and Sacchi [18]. The quantity and purity of RNA were confirmed by spectrophotometry and agarose gel electrophoresis. Reverse transcription was performed using total RNA, RNase H^- ^reverse transcriptase (Superscript II, Gibco BRL, Life Technologies, Gaithersburg, MD) and random primer hexamers.

### cDNA microarray

cDNA microarray data was obtained using a Human UniGene 1 Microarray (IncyteGenomics, St. Louis, Missouri, USA) that contains a set of 9128 clones corresponding to 8524 unique genes/EST clusters. The analysis has been performed on pooled RNA extracted by PBMC pellet of three ATRX patients in comparison to that obtained from a pool of 42 normal males (7.6 ± 2.4 years). The results were analyzed with the GEMTOOLS Analysis Software (Incyte Genomics). Raw and processed cDNA microarray data have been submitted to public repository: "Gene Expression Omnibus-GEO" http://www.ncbi.nlm.nih.gov/geo with the accession number GSE22028.

### Separation of reticulocytes and immortalized lymphoblasts of control and pathological subjects

Approximately 10 ml peripheral blood in heparin were obtained from controls and patients. Peripheral blood samples were washed 3 times in reticulocyte saline buffer (4°C) (RS: 1.3 M NaCl, 0.05 M KCl, 0.074 M MgCl_2_.6H_2_O pH 7.4) and then centrifuged at 10000 g for 20 minutes at 4°C and 0.5 ml was removed from the reticulocyte-rich top layer. Reticulocytes were concentrated by centrifugation at 3500 rpm for 30 min at 4°C. The supernatant was removed, and the top 0.3-0.5 ml reticulocyte-rich fraction was added to 2 ml of ice-cold RS and layered on a column consisting of 10 ml of 2 parts cellulose (C-8002, Sigma-Aldrich, Steinheim, Germany) and 1 part sigmacell type 50 microcrystallin cellulose (S-5504, Sigma-Aldrich, Steinheim, Germany) in RS. The red cells were eluted by centrifuging the column at 1200 rpm at 4°C for 2 min. The eluate was washed three times in 10 ml of ice-cold PBS and then the reticulocyte enriched fraction was recovered by centrifugation and resuspended in 2 ml of ice-cold PBS. RNA was extracted with TRI reagent (T-9424, Sigma-Aldrich, Steinheim, Germany). Lymphoblasts from EBV cell lines of controls (Immort CTRLs) and patients (Immort ATRX-1 and ATRX-2) were maintained in RPMI-1640 (Gibco, Invitrogen, Scotland, UK) media supplemented with 10% (vol/vol) heat-inactivated fetal bovine serum and penicillin-streptomycin (50 units/50 μg/ml). The cell cultures were incubated at 37°C in a humidified 5% CO2 incubator and the culture medium was changed twice a week.

### Human brain tissues and non cerebral RNAs

Brain tissue from five human subjects were studied: four males (ages 18, 26, 46, and 61) and one female (age 22) with postmortem interval between 4 and 36 hours. The specimens had been obtained at autopsy from the Forensic Medicine Department at the Karolinska Institute under guidelines approved by the ethics committee and the Swedish Board of Health and Social Welfare. The specimens were frozen in dry ice-cooled isopentane and stored at -80°C. We have only access to blocks of tissue from selected areas (spinal cord, medulla oblongata, cerebellum, striatum, hippocampus and neocortex). Total RNA was extracted as described by Chomczynski and Sacchi [18].

The human non cerebral total RNAs were commercially obtained from Ambion.Inc (Austin, TX): pancreas (cod.7954); liver (cod.7960); heart (cod.7966); lung (cod.7968); spleen (cod.7970); testis (cod.7972); ovary gland (cod.7974); kidney (cod.7976); skeletel muscle (cod.7982).

### mRNA splice variants by PCR analysis

To detect GRAF1/OPHN-1-L alternative transcripts were used the following primers: F19: CCGATATGCCTCTCACCAAT; F18: GCAGCCATCATGGACATCAA; R22: AGACCGTGCCTGCTGTGAAC; R22b: CATTATCGAAGACCGTGCCTG. Amplification were performed under the following conditions: denaturation step at 95°C for 3 min; 95°C × 1 min, 67°C for F19-R22 or 60°C for F18-R22b × 2 min, 72°C × 3 min × 35 cycles. Amplification products were separated by 1,8% agarose gel electrophoresis and visualized with ethidium bromide. To confirm the identify of alternative amplified variants, DNA fragments were recovered by glass-milk method [19] and sequencing was performed by standard fluorescent dideoxy chain-termination procedure with the Abi Prism 377 automatic sequencer.

### Quantitative Real Time RT-PCR

Quantitative Real Time RT-PCR (qRT-PCR) experiments were performed in the ABI PRISM 7700 Sequence Detection System from Applied Biosystems. Three sequence-specific oligonucleotides were designed using the Primer Express oligo design software (Applied Biosystems) based on the sequence of target gene. The following genes were analyzed: human Oligophrenin-1 (OPHN-1, GenBank ID: NM_002547, clone ID: 4216520), transcriptional regulator ATRX isoform-1 (XNP/ATRX, GenBank ID: NM_000489), GRAF1/Oligophrenin-1-like (GRAF1/OPHN-1-L, GenBank ID: NM_015071), Alpha hemoglobin (GenBank ID: NM_000558), Beta hemoglobin (GenBank ID: NM_000518), HAIK1 type I, intermediate filament cytocheratin or human Keratin 23 gene (HAIK1 or KRT23, clone ID: 2820372), putative lymphocyte G0/G1 switch or G0S2 gene (G0/G1 or G0S2, GenBank ID: BE873759,clone ID 1217764).

Primers and probes used for qRT-PCR are reported in Additional file 1 Table S1. Details of the conditions used for qRT-PCR are previously described [20].

## Results

### Identification of the genes whose expression is altered in ATRX peripheral blood cells

cDNA microarray results showed that only a limited number of genes was significantly affected in the ATRX syndrome. Out of 8524 transcripts analyzed 35 decreased (ratio ATRX/control < 0.5; Table 1) while 25 increased (ratio ATRX/control > 2; Table 2).

**Table 1:** List of the first 35 decreased transcripts revealed by cDNA microarray analysis (ratio ATRX/control < 0.5).

	Gene name	ratio ATRX/control	GenBank ID	Clone ID
1	putative lymphocyte G0/G1 switch or G0S2 gene	0.23	BE873759	1217764
2	defensin, alpha 1, myeloid-related sequence	0.24	M26602	1716669
3	defensin, alpha 5, Paneth cell-specific	0.25	NM_021010	4174437
4	HAIK1 type I, intermediate filament cytocheratin or human Keratin 23 gene (KRT23)	0.26	25984	2820372
5	tumor necrosis factor, alpha-induced protein 3	0.29	AI028304	1878791
6	Oligophrenin-1 (the signal is probably reflecting cross-hybridization to GRAF1/OPHN-1-L, see text)	0.30	NM_002547	4216520
7	H.sapiens DNA for cyp related pseudogene	0.30	X90579	2073646
8	ESTs	0.31	AW470039	2136337
9	DKFZP434N093 protein	0.33	BG392388	1919233
10	XIAP associated factor-1	0.33	BG541762	4738521
11	immediate early response 3	0.36	N32077	1424495
12	Homo sapiens clone 24407 mRNA sequence	0.36	AF070575	488205
13	v-jun avian sarcoma virus 17 oncogene homolog	0.37	AI078377	1969563
14	small inducible cyt. Subfam. A (Cys-Cys), member 18, pulmonary and activation-regulated	0.39	NM_002988	3473223
15	regulator of G-protein signalling 1	0.40	S59049	3120390
16	branched chain aminotransferase 1, cytosolic	0.39	AI970531	3290230
17	hypothetical protein FLJ23231	0.39	AA640102	2348706
18	small inducible cytokine subfamily B (Cys-X-Cys), member 10	0.39	NM_001565	1656473
19	lactotransferrin	0.42	M73700	1861911
20	B-cell CLL/lymphoma 3	0.43	NM_005178	1867294
21	polymerase (RNA) III (DNA directed) (39 kD)	0.43	XM_009639	1682994
22	HS (clone B3B3E13) chrom 4p16.3 DNA fragment	0.43	BE789464	2619
23	RAB6 interacting, kinesin-like (rabkinesin6)	0.43	AV714379	1975194
24	HS cDNA FLJ11918 fis, clone HEMBB1000272	0.44	AA810748	1921725
25	DEAD/H (Asp-Glu-Ala-Asp/His) box polyp. 21	0.45	U41387	1879532
26	dual specificity phosphatase 2	0.44	NM_004418	518826
27	hypothetical protein, expressed in osteoblast	0.44	F12860	2537863
28	hypothetical protein FLJ23306	0.48	AI088306	2372178
29	interleukin 8	0.48	AV717082	2785701
30	COX15 (yeast) homolog, cyt.c oxid. ass.protein	0.47	XM_005811	1976440
31	Incyte EST	0.49		2895226
32	hypoxia-ind. fact. 1, alpha sub (basic helix-loop-helix transcription factor)	0.48	AU134078	1711151
33	dihydrolipoamide branched chain transacylase (E2 comp of branched chain keto acid dehyd. complex)	0.47	NM_001918	3521142
34	Human glucocorticoid receptor alpha mRNA, variant 3' UTR	0.47	U25029	3127171
35	ATP/GTP-binding protein	0.48	AA188236	3032908

**Table 2:** List of the first 25 increased transcripts revealed by cDNA microarray analysis (ratio ATRX/control > 2).

	Gene name	ratio ATRX/control	GenBank ID	Clone ID
1	chemokine (C-X3-C) receptor 1	2.99	U20350	2305611
2	ESTs, Weakly sim to A48809 carboxylesterase [HS]	2.80	N44535	1877718
3	hemoglobin, beta	2.76	BG529867	3872557
4	granzyme B	2.69	M57888	1879670
5	hemoglobin, delta	2.66	NM_000519	133350
6	hemoglobin, gamma A	2.66	XM_006556	2156647
7	prostaglandin E receptor 4	2.62	NM_000958	1631793
8	nuclear receptor subfamily 1, group H, member 2	2.47	BE878950	2581075
9	VPS28 protein	2.37	BF663123	2355041
10	hypothetical protein FLJ23467	2.30	NM_024575	2632784
11	adenylyl cyclase-associated protein	2.17	BG286995	30672
12	selenophosphate synthetase 2	2.23	AW957160	1687542
13	granzyme A	2.16	AA283172	311922
14	CD8 antigen, alpha polypeptide (p32)	2.17	M12824	1846142
15	Fc fragment of IgE, high aff. I, rec for; alpha polyp.	2.18	BG542554	2762987
16	lamin B receptor	2.18	NM_002296	1803808
17	leukotriene A4 hydrolase	2.08	AI636026	1988019
18	Homo sapiens integrin, beta 1	2.08	BE880168	4089291
19	IQ motif containing GTPase activating protein 1	2.14	AI719158	1697314
20	CD3 D antigen, delta polypeptide (TiT3 complex)	2.11	AA310902	3297914
21	VPS28 protein	2.03	AF067420	2860815
22	Homo sapiens mRNA for single-chain antibody	1.97	AL560682	2237234
23	Homo sapiens clone 24775 mRNA sequence	1.99	AA402981	1671596
24	T cell receptor gamma locus	2.03	AI972955	1857652
25	vinculin	2.00	NM_014000	999864

**Figure 1:**
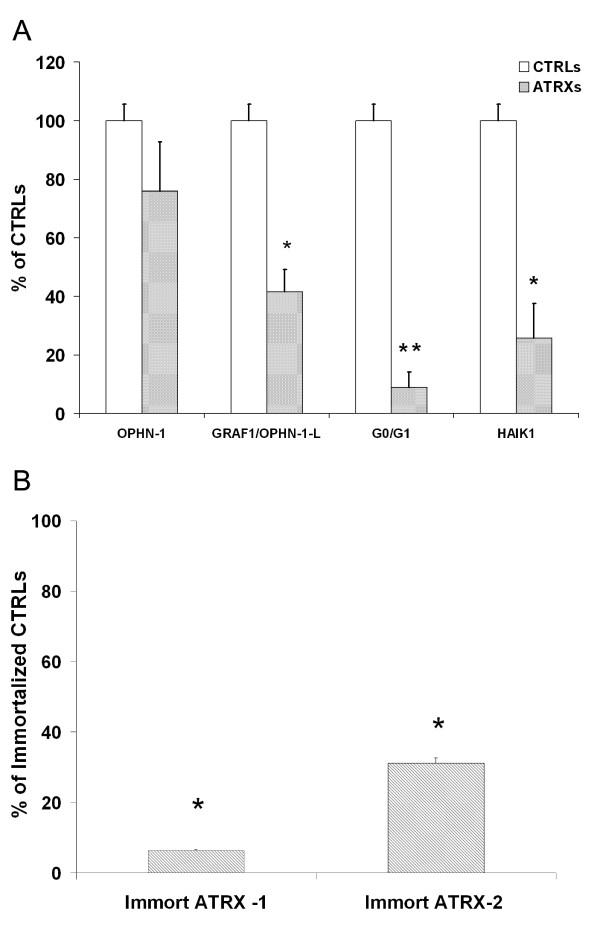
**Quantitative mRNA expression in ATRX patients**. **A**. qRT-PCR analysis of the OPHN-1, GRAF1/OPHN-1-L, G0/G1 and HAIK1 transcript levels in ATRX patients (n = 3, ATRX-1, ATRX-2, ATRX-3) and in normal controls (n = 8). Results are expressed as percentage of control average. Each bar represents the mean ± SEM. Statistical significance was determined by Student t test and indicated by asterisks: * p < 0.05; ** p < 0.01. **B**. qRT-PCR analysis showing a reduction of the GRAF1/OPHN-1-L transcript levels in the immortalized cell lines belonging to two ATRX patients, ATRX-1, ATRX-2. Results are expressed as percentage of control average (n = 4 immortalized cell lines from normal subjects). Each column is the average of three determination ± SD: * p < 0.01.

In order to confirm the results obtained by cDNA microarray analysis, we performed qRT-PCR assays for some transcripts among those showing the largest changes in gene expression (see in Table 1 ranking genes down regulated in ATRX according to the ratio ATRX/control). As shown in Figure 1 the putative lymphocyte G0/G1 switch (or G0S2) gene mRNA levels (position 1 in Table 1), and the incyte EST clone ID 2820372, HAIK1 type I, intermediate filament cytocheratin or human Keratin 23 gene (KRT23, GeneID: 25984, position 4 in Table 1) were decreased in the freshly separated lymphomonocytes obtained by three different ATRX patients confirming the cDNA microarray data. On the contrary, the quantitative analysis in qRT-PCR has not confirmed the strong reduction of oligophrenin-1 (OPHN-1) mRNA (3.3 times lower in ATRX subjects) observed by cDNA microarray (Figure 1A). However, cDNA microarray data can be influenced by other transcripts with a high level of homology. With this in mind, a bioinformatics search of the OPHN-1 gene homologs has been executed. Such analysis revealed the presence of an "Oligophrenin-1-like (OPHN-1-L)" transcript deriving from the gene encoding for a previously identified "RhoGAP" protein, called "GRAF1" (GTPase regulator associated with the focal adhesion kinase pp125FAK or Rho-type GTPase-activating protein 26) [21]. The probe sequence in the microarray (Incyte clone ID 4216520) covers part of the 5' coding sequence of the oligophrenin sequence which is 65% homologue to GRAF1, a value that is enough to produce an hybridization signal. Indeed, by quantitative RT-PCR OPHN-1 mRNA levels in PBM were one order of magnitude lower than GRAF1 levels, suggesting that the latter transcript was the major determinant of the hybridization signal.

RNA from control subjects and ATRX patients was examined by qRT-PCR analysis in order to evaluate the level of GRAF1/OPHN-1-L transcript. These results confirmed a reduction of the "GRAF1/OPHN-1-L" transcript levels with values equal to 41.64% (SEM 7.48) of the controls in the three examined subjects (Figure 1A).

In order to confirm the observed transcript variations in PBM cells we examined the GRAF1/OPHN-1-L expression in immortalized lymphocyte lines of normal and ATRX subjects. As it can be observed in Figure 1B the GRAF1/OPHN-1-L mRNA levels were drastically reduced in ATRX immortalized lines compared to immortalized control cells. OPHN-1 mRNA levels were not detectable in both ATRX and control cell lines (no amplification to 40 cycles in 90% of the analyzed samples, data not shown).

### Identification of a new splicing GRAF1/oligophrenin-1-like transcript, variant-3

The search in the database, using as query sequence *"oligophrenin-1" (*accession number NM_002547*) *supplied 2 sequences: the nucleotide sequence "GRAF1/OPHN-1-L, variant-1"(deposited with accession number NM_015071) and the nucleotide sequence "GRAF1/OPHN-1-L, variant-2" (accession number NM_001135608).

**Figure 2:**
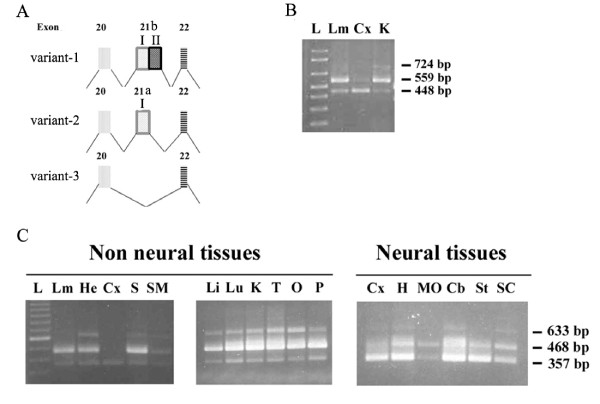
**Drawing and tissue distribution of GRAF1/OPHN-1-L isoforms**. **A**. Schematic representation of the exon 20-to-22 of GRAF1/OPHN-1-L variant-1 -2 and -3. Variant-1 contains the whole exon 21 reported in NCBI GenBank as exon 21b, variant-2 has exon 21-I (reported in NCBI GenBank as exon 21a) while exclusion of exon 21 results in the variant-3. **B**. RT-PCR amplification of the GRAF1/OPHN-1-L transcripts (variant-1: 724 bp; variant-2: 559 bp; variant-3: 448 bp) was confirmed in neural e non neural tissues (Lm: lymphomonocytes, Cx: cerebral cortex, K: kidney) with the other following primers: F18-R22b. **C**. Tissue distribution of GRAF1/OPHN-1-L isoforms. RT-PCR amplification of the GRAF1/OPHN-1-L transcripts (variant-1: 633 bp; variant-2: 468 bp; variant-3: 357 bp) from various human organs and tissues with following primers: F19-R22. L: 100 bp ladder; Lm: lymphomonocytes, He: heart, Cx: cerebral cortex, S: spleen, SM: skeletal muscle, Li: liver, Lu: lung, K: kidney, T: testis, O: ovary, P: pancreas, H: hippocampus, MO: medulla oblongata, Cb: cerebellum, St: striatum, SC: spinal cord.

The comparison of the two transcript isoforms of "GRAF1/OPHN-1-L" sequences demonstrated that they were two splicing variants of the same gene (Additional file 2 Figure S1). The "GRAF1/OPHN-1-L" gene, localized on chromosome 5q31, is composed by 23 exons and the complete translation produces a protein of 814 amino acids corresponding to the sequence deposited as "GRAF1/OPHN-1-L, variant-1". In the "GRAF1/OPHN-1-L, variant-2" is present only a 5'segment of exon 21, while the "GRAF1/OPHN-1-L, variant-1" sequence contains the whole exon 21. Therefore, the whole exon 21, of 276 nucleotides, could be subdivided in two fragments: the exon 21-I (from nucleotide position 2024 to 2134 in the sequence with accession number NM_015071: 111 bases encoding a 37-amino acids sequence) and the exon 21-II (from the nucleotide position 2135 to 2299: 165 bases encoding a 55-amino acids sequence). The transcript variant-1 contains the whole exon 21 (exon21-I + exon21-II encoding a sequence of 92 amino acids; exon 21 is reported in NCBI GenBank as exon 21b), while only exon 21-I (reported in NCBI GenBank as exon 21a) is present in the transcript variant-2 (Figure 2A, Table 3, Additional file 2 Figure S1).

**Table 3:** List of three GRAF1/OPHN-1-L isoforms: variant-1, variant-2 and variant-3.

Name	Number of exons	GenBank ID	Exon number involved in splice site (number of base involved)	Total number of aminoacids
GRAF1/OPHN-1-L, variant-1	23	NM_015071	Exon 21b 21-I + 21-II (2024-2299) 276 nt	814

GRAF1/OPHN-1-L, variant-2	23	NM_001135608	Exon21a 21-I (2024-2134) 111 nt	759

GRAF1/OPHN-1-L, variant-3	22	HM037040	No Exon 21	641

The splicing of the exon 21-II does not modify the reading frame, so that variant-2 differs from variant-1 only for the lack of 55 amino acids close to the carboxy-terminal portion. Moreover the exon 21-II contains the canonical splice acceptor and donor sites gt...ag in the correct positions (Additional file 2 Figure S1). To investigate the presence of GRAF1/OPHN-1-L transcripts a RT-PCR analysis was performed using specific primers to amplify the zone including exon 21. In particular two forward primers (F18, F19), localized to the exon 18 and 19, and two reverse primers (R22 and R22b), localized to the exon 22, have been synthetized. RT-PCR amplification with the primers F18 and R22b, generated 3 amplification bands of 724 bp, 559 bp, and 448 bp (Figure 2B). RT-PCR analysis, performed with the primers F19 and R22 generated 3 amplification bands of 633 bp, 468 bp, and 357 bp (Figure 2C). The sequencing of the amplification products revealed that the bands of greater size (724 bp, 633 bp) contained the whole exon 21 (21-I + 21-II), the intermediate bands (559 bp, 468 bp) contained only exon 21-I, while the band of smaller size (448 bp, 357 bp) did not contain exon 21. Therefore, besides the two known transcripts (variant-1 and variant-2), one additional transcript variant (variant-3) was detected in such analysis, suggesting that GRAF1/OPHN-1-L gene generated three alternatively spliced transcripts differing in the use of exon 21 (Additional file 2 Figure S1). The translation of variant-3 sequence produces a protein of 641 amino acids. The sequence of variant-3 has been submitted to NCBI GenBank with accession number HM037040.

### Tissue distribution of "GRAF1/oligophrenin-1-like" isoforms

GRAF1/OPHN-1-L expression was examined in specific brain regions, as well as in a series of other adult tissues. Figure 2C shows the tissue distribution of the GRAF1/OPHN-1-L variants obtained using the forward primer F19, localized to exon 19, and the reverse primers R22, localized to exon 22. The GRAF1/OPHN-1-L variant-3 was predominantly and highly expressed in the human cerebral areas (357 bp, Figure 2C). In the adult brain, the transcript variant-3 was expressed in all tested regions including cerebral cortex, hippocampus, medulla oblongata, cerebellum, striatum, and spinal cord. In parenchymatous organs, endocrine glands, muscular tissue and lymphomonocytes the variant-2 is predominant (468 bp, Figure 2C). On the contrary the variant-1 is weakly expressed in the cerebral tissues and in the peripheral lymphocytes.

### mRNA levels for alpha-and beta-globins in reticulocytes

Since alpha-thalassemia is one of the main feature of the ATRX syndrome, changes in expression of the globin genes were also investigated. Probes for alpha-globin chains were not present in the cDNA microarray platform used for the present study, but we could detect significant increases in the levels of the beta, gamma and delta globin mRNAs in ATRX patients (values 2.76, 2.70, 2.66 times higher respectively, see Table 2). The same analysis did not reveal significant differences for the embryonic zeta-globin chains.

**Figure 3:**
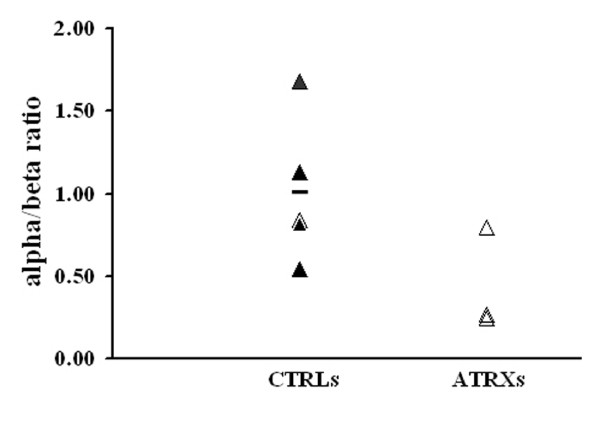
**Alpha/beta ratio in ATRX lymphomonocytes**. Alpha-globin/beta-globin mRNA ratio measured by qRT-PCR in lymphomonocytes from the peripheral blood of patients, ATRX-1, ATRX-2, ATRX-3 compared to control subjects (CTRLs).

In order to confirm and to extend the cDNA microarray results we performed a qRT-PCR analysis for the alpha-and beta-globin transcripts. As reported in Figure 3 we found a reduction of the alpha/beta ratio (alpha globin mRNA/beta globin mRNA) in two ATRX patients (ATRX-1 and ATRX-2) with values of 0.26 and 0.24 (controls mean = 1 ± 0.43; Figure 3), while ATRX-3 had a value in the range of the controls (ATRX-3, alpha/beta ratio 0.78). It is well-known that hematologic findings vary widely among ATRX patients and in some cases the manifestation of alpha-thalassemia may be subtle [22]. Indeed, alpha/beta transcript ratios are in agreement with the presence of erythrocyte HbH inclusion bodies in ATRX-1 and ATRX-2 but not in ATRX-3. The percentage of HbH cells to normal RBCs, visualized by staining with 1% brilliant cresyl blue, were 1%, 3.7% and 0% in ATRX-1, ATRX-2 and ATRX-3, respectively.

**Figure 4:**
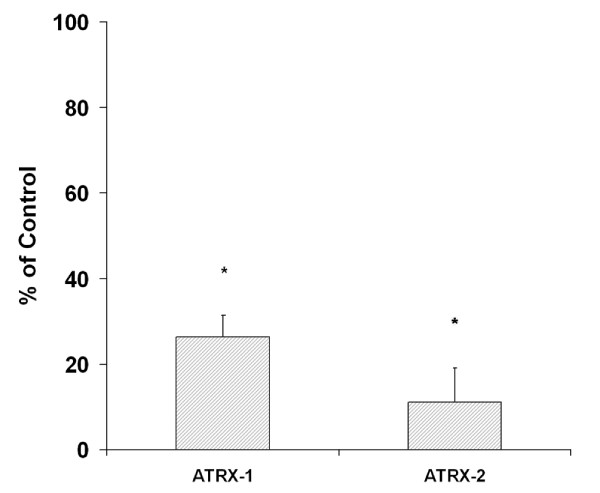
**Alpha/beta ratio in ATRX reticulocytes**. qRT-PCR analysis showing a reduction of alpha/beta ratio in reticulocytes purified from the peripheral blood of patients (ATRX-1 and ATRX-2) compared to control (pooled samples of six normal subjects). Data are expressed as % of control and represent mean ± SD of two independent experiments. Statistical significance was determined by Student t test and indicated by asterisk: * p < 0.05.

The presence of detectable globin transcripts in RNAs extracted from lymphomonocyte samples suggested that these cell preparations contained a nonnegligible amount of contaminating reticulocytes. This hypothesis is supported by the observation that such transcripts showed a 3 times reduction in samples enriched for CD4+ lymphocytes and by the fact they were not detectable in RNA samples extracted by immortalized lymphocyte cell lines. On this basis, we decided to perform a quantitative analysis of alpha-and beta-globin transcripts in RNA samples extracted from reticulocytes isolated from the peripheral blood of control and ATRX subjects. Indeed, such analysis confirmed the reduction of the alpha/beta ratio in the ATRX subjects (Figure 4). However the absolute values of the alpha-and beta-globin transcripts showed remarkable variations between samples and did not allow to establish if the modification of the ratio is due to a decrease of alpha-globin transcripts and/or an increase of beta-globin ones.

## Discussion

It is well-known that one of the main clinical features of the syndrome is the presence of a decreased expression of alpha-globin genes leading to a form of alpha thalassemia. Indeed, qRT-PCR analysis confirmed a decrease of the alpha-globin mRNA/beta-globin mRNA ratios, in agreement with the known molecular alterations of the disease. Moreover, the increase in beta-like chain gene transcription, observed in microarray analysis, could be considered a compensatory activity to alpha chain deficiency. However much less is known about the mechanisms responsible for the other dominant clinical feature, the mental retardation. Many lines of evidence support a role for ATRX protein during cerebral development and Berube et al. [23] proposed that transcription-dependent events regulated by ATRX play a critical role in mediating the survival of neurons in the developing cortex and hippocampus. To date, although the ATRX protein structure suggests a role in chromatin regulation, it is not clear whether ATRX imparts predominantly a blocking or a positive effect on gene expression.

The complexity of the syndrome suggests that ATRX protein could be involved in the regulation of various unidentified genes and the present work shows, indeed, that altered expression in specific genes can be found in this disease. As an exploratory tools to identify potential candidate genes we used a cDNA array analysis, that does not cover the entire range of cellular transcripts. Indeed, such analysis suggested some specific transcripts whose changes were confirmed by quantitative RT-PCR analysis (a putative lymphocyte G0/G1 switch or G0S2 gene [24,25] and the human Keratin 23 gene, HAIK1 [26]. On the other hand, the gene expression change for oligophrenin-1 was not confirmed by RT-PCR but drew our attention on an homologous gene, the GRAF1/OPHN-1-L gene, localized at chromosome 5q31 and encoding for a Rho-GTPase-activating protein (Rho-GAP). Indeed, GRAF1/OPHN-1-L expression is decreased both in blood mononuclear cells and in immortalized lymphoblastoid cells of ATRX patients. Although, our analysis has been performed in peripheral blood lymphomonocytes, it is interesting that the GRAF1/OPHN-1-L transcript is highly expressed in the human brain [27] and homologous to a gene, OPHN-1, previously associated with X-linked mental retardation [28]. GRAF1/OPHN-1-L protein contains a centrally located GAP domain followed by a serine/proline-rich domain and a carboxyl-terminal SH3 domain. The SH3 domain was shown to specifically bind to a proline-rich region in the carboxy-terminus of FAK, protein-tyrosine kinase associated with focal adhesions. Hildebrand et al. [29] reported that, in vitro, GRAF1/OPHN-1-L has GAP activity for Cdc42 and RhoA. Taylor et al. [27,30] showed that GRAF1/OPHN-1-L specifically regulates Rho activity *in vivo*, down-regulating Rho activity in Swiss 3T3 cells but enhancing Rho-dependent effects in PC12 cells. The latter cell line, used primarily as a neuron model, has been established from a rat pheochromocytoma, expressing high level of GRAF1/OPHN-1-L, while Swiss 3T3 cells (or other fibroblast cell lines) did not express detectable levels [30]. Moreover, GRAF1/OPHN-1-L is enriched in the brain, where it could regulate Rho-mediated neurite retraction, and it has been suggested that GRAF1/OPHN-1-L might play an important role in neuronal cell morphology [27,30]. In particular GRAF1/OPHN-1-L should function as mediator linking the extracellular guiding signals to the intracellular signal transduction pathways that are important for neuronal morphogenesis as well as for cytoskeletal dynamics within neuronal growth cones [27]. Alteration of such pathways influencing growth and guidance of axon outgrowth at neuronal growth cones might lead to impaired formation of brain structures.

In the present paper, we report the tissue distribution of a novel alternative splicing transcript (variant-3), lacking the entire exon 21, and show that it represents the main transcript in the brain. Exon 21 encodes for a portion of GRAF1/OPHN-1-L protein (from aminoacid 664 to 755 in variant-1) localized between the serine/proline rich domain (from aminoacid 584 to 701) and a carboxy-terminal Src-homology 3 (SH3) domain (from aminoacid 756 to 814). The three isoforms represent tissue-specific alternative products. It is possible to suggest that occurrence of exon 21 might be of importance to regulate the distance between the serine/proline rich domain and SH3 domain and the composition of the serine/proline rich domain.

Another issue for future investigations is suggested by the observation that somatic mutations of ATRX are associated with alpha thalassaemia myelodysplastic syndrome, an acquired form of alpha-thalassaemia that most commonly arises in the context of myelodysplasia [31,32]. Taking into consideration that GRAF1/OPHN-1-L inactivation has been detected in myelodysplasias and leukemias [21,33], suggesting a role as tumor suppressor for this gene, the link between inactivating mutations of ATRX and decreased expression of GRAF1/OPHN-1-L, observed in the present work, might explain the selection of ATRX mutations in the course of leukemic progression. Analysis of GRAF1/OPHN-1-L expression and correlation with ATRX activity deserves further studies in such hematological malignancies.

## Conclusion

Our work identifies some of the transcripts showing altered expression in ATRX syndrome in peripheral blood mononuclear cells and stimulates further studies aimed to the direct measurements of such changes in brain tissues of patients affected by this rare disease. Moreover, these data provide the rationale for investigations aimed to analyse the possible involvement of GRAF1/OPHN-1-L gene in other forms of mental retardation.

## Competing interests

The authors declare that they have no competing interests.

## Authors' contributions

VB, AR, MF and DFC  participated in the conception and design of the study, in the acquisition and analysis of data, and drafted and revised the article; NM, LC, GR, ST, TM participated in the acquisition of molecular data and revised of the article; CR and GC participated in the acquisition and analysis of clinical data and revised the article. All authors read and approved the final manuscript.

## Pre-publication history

The pre-publication history for this paper can be accessed here:

http://www.biomedcentral.com/1755-8794/3/28/prepub

## Supplementary Material

Additional file 1 Table S1: Primers and probes used for qRT-PCR. Click here for file

Additional file 2 Figure S1: Sequence alignment of the GRAF1/OPHN-1-L isoforms: variant-1, -2 and -3. Click here for file
