# Supplementary material for: Decreased expression of GRAF1/OPHN-1-L in the X-linked alpha thalassemia mental retardation syndrome
Source: BMC Med Genomics. 2010 Jul 6;3:28. doi: 10.1186/1755-8794-3-28 (PMC2915949; doi:10.1186/1755-8794-3-28)
Supplement: Additional file 1 — Table S1: Primers and probes used for qRT-PCR. [file 1755-8794-3-28-S1.DOC]

**Table S1. Primers and probes used for qRT-PCR**

| **Gene Name** | **Forward primer 5’…3’** | **Fluorogenic probe**  **5’ FAM-….. TAMRA 3’** | **Reverse primer 5’…3’** |
| --- | --- | --- | --- |
| **OPHN-1** | AGACCAAAGGGATCAAGACAGAA | TGTACCGCACTGTGGGCAGCAATAT | CCAGTCACTATTATGAAAATCAACATCT |
| **XNP/ATRX** | TAAAGGGCCTGAATTTAGAAGCA | AAAAAACGCGGAGAAGATGGGCTTCA | CACAAGCAGTGCAGCTCACA |
| **Alpha-globin** | GCACGCTGGCGAGTATGG | CGGAGGCCCTGGAGAGGATGTTC | TCGAAGTGCGGGAAGTAGGT |
| **Beta-globin** | GCACGTGGATCCTGAGAACTT | AGGCTCCTGGGCAACGTGCTG | TGATGGGCCAGCACACAGA |
| **GRAF1/OPHN-1-L** | GCATTGGCTTCAGCATAATCAG | TGGAAACCAGAGGGATCAACGAGCA | CCATCAGGACACTCAGCAACTTC |
| **HAIK1** | GAGGATATGAGACAAGAATATGAGCTTATAAT | ACTTGGACACTTGGTATAAAGAACAGTCTGCAGC | GGCTGCCTCCTGGGACAT |
| **Putative G0/G1 switch gene** | CATCACATGCTGAGCTGTAGCA | ACCACTGAGACTACGAACCTCTGTCCTCTGAAGA | CTCAGAACAGTGGCCCACACT |
