# Supplementary material for: Decreased expression of GRAF1/OPHN-1-L in the X-linked alpha thalassemia mental retardation syndrome
Source: BMC Med Genomics. 2010 Jul 6;3:28. doi: 10.1186/1755-8794-3-28 (PMC2915949; doi:10.1186/1755-8794-3-28)
Supplement: Additional file 2 — Figure S1: Sequence alignment of the GRAF1/OPHN-1-L isoforms: variant-1, -2 and -3. [file 1755-8794-3-28-S2.DOC]

GRAF/OPHN-1-L var1 AGCTTACAGCCCAACATGAACTCCAGTGACCCAGACCTGGCTGTGGTCAAACCCACCCGG 2012

GRAF/OPHN-1-L var1 S L Q P N M N S S D P D L A V V K P T R

GRAF/OPHN-1-L var2 AGCTTACAGCCCAACATGAACTCCAGTGACCCAGACCTGGCTGTGGTCAAACCCACCCGG 2012

GRAF/OPHN-1-L var2 S L Q P N M N S S D P D L A V V K P T R

GRAF/OPHN-1-L var3 AGCTTACAGCCCAACATGAACTCCAGTGACCCAGACCTGGCTGTGGTCAAACCCACCCGG 2012

GRAF/OPHN-1-L var3 S L Q P N M N S S D P D L A V V K P T R

GRAF/OPHN-1-L var1 CCCAACTCACTCCCCCCGAATCCAAGCCCAACTTCACCCCTCTCGCCATCTTGGCCCATG 2072

GRAF/OPHN-1-L var1 P N S L P P N P S P T S P L S P S W P M

GRAF/OPHN-1-L var2 CCCAACTCACTCCCCCCGAATCCAAGCCCAACTTCACCCCTCTCGCCATCTTGGCCCATG 2072

GRAF/OPHN-1-L var2 P N S L P P N P S P T S P L S P S W P M

GRAF/OPHN-1-L var3 CCCAACTCACT------------------------------------------------- **2023**

GRAF/OPHN-1-L var3 P N S L-------------------------------------------------

GRAF/OPHN-1-L var1 TTCTCGGCGCCATCCAGCCCTATGCCCACCTCATCCACGTCCAGCGACTCATCCCCCGTC 2132

GRAF/OPHN-1-L var1 F S A P S S P M P T S S T S S D S S P V

GRAF/OPHN-1-L var2 TTCTCGGCACCATCCAGCCCTATGCCCACCTCATCCACGTCCAGCGACTCATCCCCCGTC 2132

GRAF/OPHN-1-L var2 F S A P S S P M P T S S T S S D S S P V

GRAF/OPHN-1-L var3 ------------------------------------------------------------ **2023**

GRAF/OPHN-1-L var3 ------------------------------------------------------------

GRAF/OPHN-1-L var1 AGgtctgttgcagggtttgtttggttttctgttgctgccgttgttctctcattggctcgg 2192

GRAF/OPHN-1-L var1 R S V A G F V W F S V A A V V L S L A R

GRAF/OPHN-1-L var2 AG---------------------------------------------------------- **2134**

GRAF/OPHN-1-L var2 S----------------------------------------------------------

GRAF/OPHN-1-L var3 ------------------------------------------------------------ 2023

GRAF/OPHN-1-L var3 ------------------------------------------------------------

GRAF/OPHN-1-L var1 tcctctcttcatgcagtgttcagcctcctcgtcaactttgttccctgccatccaaacctg 2252

GRAF/OPHN-1-L var1 S S L H A V F S L L V N F V P C H P N L

GRAF/OPHN-1-L var2 ----------------------------------------------------------- 2134

GRAF/OPHN-1-L var2 -----------------------------------------------------------

GRAF/OPHN-1-L var3 ----------------------------------------------------------- 2023

GRAF/OPHN-1-L var3 -----------------------------------------------------------

GRAF/OPHN-1-L var1 cacttgctttttgacaggccagaagaagcggtacatgaagactccagCACACCGTTCCGG 2312

GRAF/OPHN-1-L var1 H L L F D R P E E A V H E D S S T P F R

GRAF/OPHN-1-L var2 -----------------------------------------------CACACCGTTCCGG 2147

GRAF/OPHN-1-L var2 ---------------------------------------------- T P F R

GRAF/OPHN-1-L var3 -----------------------------------------------CACACCGTTCCGG 2036

GRAF/OPHN-1-L var3 ----------------------------------------------- T P F R

**Figure S1. Sequence alignment of the GRAF1/OPHN-1-L isoforms: variant-1, -2 and -3**. The sequence of whole exon 21 (reported in NCBI GenBank as exon 21b, from nucleotide 2024 to 2299) is indicated in blue. The sequence of exon 21-I (reported in NCBI GenBank as exon 21a) is from nucleotide 2024 to 2134. The donor and acceptor splicing sites, gt..ag, are indicated in red. GRAF1/OPHN-1-L “variant-1” and “variant-2” have GenBank accession number: NM_015071 and NM_001135608, respectively. The adopted numbering is referred to the sequence NM_015071. The sequence of variant-3, identified in the present study, has been submitted to NCBI GenBank with accession number HM037040. The lower sequence represents the derived amino acid translation product of GRAF1/OPHN-1-L.
